# Editorial: Epigenetic and molecular control of development and germ cell fate determination

**DOI:** 10.3389/fcell.2023.1274602

**Published:** 2023-08-29

**Authors:** Kui Liu, Hengbin Wang

**Affiliations:** ^1^ Department of Obstetrics and Gynecology, Li Ka Shing Faculty of Medicine, The University of Hong Kong, Pokfulam, Hong Kong SAR, China; ^2^ Shenzhen Key Laboratory of Fertility Regulation, Center of Assisted Reproduction and Embryology, The University of Hong Kong-Shenzhen Hospital, Shenzhen, Guangdong, China; ^3^ Division of Hematology, Oncology and Palliative Care, Department of Internal Medicine, Massey Comprehensive Cancer Center, Virginia Commonwealth University, Richmond, VA, United States

**Keywords:** epigenetics, genetics, molecular, germ cell, fate determination, development

Genetics has long been considered the cornerstone of development, but the role of epigenetics has been highlighted recently. “Epi”genetics, the mechanism regulating gene expression beyond genetics, influences development by integrating the environment and developmental signals to fine-tune cellular gene expression programs and determine cell fate. As an important aspect of mammalian development, the production of germ cells from gametes is also strictly controlled by genetic and epigenetic mechanisms. This Research Topic, focusing on the genetic and epigenetic mechanisms controlling development and game cell production, comprises six articles: two on female germ cells, two on male germ cells, one on embryonic stem cell (ESC) fate regulation, and one on early embryonic development.

Polycomb group (PcG) proteins are a conserved family of proteins that help maintain the repressed status of many master developmental genes throughout development (Aloia et al., 2013; Blackledge and Klose, 2021). Two major Polycomb Repressive Complexes were reported: PRC1 and PRC2. PRC1 consists of core subunits Ring1, Ring2, and Bmi1 and is responsible for histone H2A lysine 119 ubiquitination (H2AK119ub) (Wang et al., 2004). PCGF2, also known as Mel18, can replace Bmi1 in PRC1 for H2AK119ub when phosphorylated (Elderkin et al., 2007). Zhang Y. et al. investigated the role of pcgf2 in folliculogenesis and ovulation by generating granulosa cell-specific pcfg2 knockout mice. These mice exhibit loss of follicle, defects in ovulation, prolonged estrus cycle, and infertility in a subgroup of mice. These defects were attributed to pcfg2-regulated progesterone receptor (a key ovulation gene) expression through H2AK119ub. In the review article by Wu G. et al., the authors summarized recent advances in studying *in vitro* differentiation of ovarian follicles from pluripotent stem cells, including progress in the generation of primordial germ cell-like cells, pre-granulosa cells, and theca cells *in vitro* from stem cells.

During the pachytene stage in mammalian meiosis, the X and Y chromosomes remain largely unsynapsed and were wrapped into a structure called the “sex body” (Solari, 1974; Turner, 2015). Several proteins, including the DNA damage response factors, the downstream Fanconi anemia proteins, and canonical repressive histone modifications, have been reported in the sex body, yet the formation, structure, and function of this special functional domain remain largely unknown. Li reviewed the reported factors associated with the sex body, aiming to provide new insights to study this subcellular structure. MicroRNAs (miRNAs) have been shown to play essential roles in mammalian spermatogenesis (Chen et al., 2017). In the review article by Chen and Han, the authors summarized literature on miRNAs in mammalian spermatogenesis using *in vivo* genetic methods and suggested new approaches using innovative technologies.

ESC hold great promise for regenerative medicine given their ability of self-renewal during *in vitro* culture and their potential to differentiate into virtually all cell types (Mahla, 2016). Transcription, which governs cell gene expression profiles and defines cell identity, is regulated at multiple steps, including elongation (Cramer, 2019). Wang X. et al. reviewed recent advances on understanding the regulation of transcription elongation by transcription factors and epigenetic modifications on ESC fate determination. In previous studies, Remodeling and Spacing Factor 1 (RSF1) was identified as a reader of H2AK119ub (Zhang et al., 2017). In the study carried out by Parast et al., *Xenopus* RSF1 (rsf1) was found to regulate not only gastrulation but also induce neural and neural crest defects. Their experiments further showed that a construct with deletion of the UAB domain, which is required for RSF1 to recognize the H2AK119ub nucleosomes, failed to rescue rsf1 morphant embryos and was less effective in interfering with early *Xenopus* development than the wild-type RSF1 when ectopically expressed. Thus, this study revealed a critical role of recognizing H2AK119ub in the function of RSF1 in *Xenopus* development.
